# Effects of AlN Coating Layer on High Temperature Characteristics of Langasite SAW Sensors

**DOI:** 10.3390/s16091436

**Published:** 2016-09-06

**Authors:** Lin Shu, Bin Peng, Yilin Cui, Dongdong Gong, Zhengbing Yang, Xingzhao Liu, Wanli Zhang

**Affiliations:** 1 State Key Laboratory of Electronic Thin Films and Integrated Devices, University of Electronic Science and Technology of China, Chengdu 610054, China; s89s89s@126.com (L.S.); g3ingmtg@gmail.com (Y.C.); 15196609270@163.com (D.G.); xzliu@uestc.edu.cn (X.L.); wlzhang@uestc.edu.cn (W.Z.); 2 China Gas Turbine Establishment, Jiangyou 621703, China; zbyang668@163.com

**Keywords:** langasite, AlN coating layer, high temperature, temperature coefficient of frequency

## Abstract

High temperature characteristics of langasite surface acoustic wave (SAW) devices coated with an AlN thin film have been investigated in this work. The AlN films were deposited on the prepared SAW devices by mid-frequency magnetron sputtering. The SAW devices coated with AlN films were measured from room temperature to 600 °C. The results show that the SAW devices can work up to 600 °C. The AlN coating layer can protect and improve the performance of the SAW devices at high temperature. The SAW velocity increases with increasing AlN coating layer thickness. The temperature coefficients of frequency (TCF) of the prepared SAW devices decrease with increasing thickness of AlN coating layers, while the electromechanical coupling coefficient (*K*^2^) of the SAW devices increases with increasing AlN film thickness. The *K*^2^ of the SAW devices increases by about 20% from room temperature to 600 °C. The results suggest that AlN coating layer can not only protect the SAW devices from environmental contamination, but also improve the *K*^2^ of the SAW devices.

## 1. Introduction

Nowadays, a wireless and battery-free sensor which can stably operate in harsh environments is in great demand for many industries, particularly in aerospace, automotive, and energy industries. Sensing of physical properties such as temperature, strain, and vibration are useful for dynamic and static health monitoring and maintenance of the target components. Sensors based on surface acoustic wave (SAW) technology are attractive due to its characteristics of passivity, wireless, high sensitivity, high precision, and good reproducibility. In a typical SAW device, a surface mechanical wave is generated and propagates in a piezoelectric substrate due to an electro-mechanical coupling effect. The characteristics of SAW sensors are strongly dependent on the piezoelectric substrate. For example, the operation temperature is strongly dependent on the phase transition temperature of the piezoelectric substrate. Among various piezoelectric materials, lanthanum gallium silicate (La_3_Ga_5_SiO_14_, LGS，langasite) has been thought as one of the most attractive materials in high temperature application due to the absence of a phase transition from room temperature up to its melting temperature (1450 °C), good temperature stability, and large coupling constant K^2^ (0.34%), which is two times larger than that of ST-quartz (0.14%) [1]. Peng [2] and Thiele [3] fabricated a high temperature SAW gas sensor with LGS materials. Another LGS SAW gas sensor for harsh environments was reported by Greve et al. [4]. We also reported SAW strain sensors based on different cuts of LGS substrate at high temperature [5].

It was found that an obvious signal attenuation or an unexpected shift of the resonance frequency of the SAW sensor appears after long-term measurement at high temperature [6]. This is caused not only by the degradation of the metal electrodes, but also surface contamination in harsh environments [7]. So, a protective layer is required to improve the stability of the SAW sensor. Some works about protective coatings for SAW sensors have been reported by Cunha [8] and Freudenberg [9]. However, to the best of our knowledge, there are no reports on the thickness effects of the coatings on the performance of SAW sensors.

In this paper, SAW sensors based on an LGS substrate with Euler angles of (0°, 138.5°, 26.6°) are fabricated. AlN thin film is deposited on the entire surface of the SAW devices as a protective layer. We chose AlN film as a protective layer on SAW devices due to its excellent properties, such as high hardness (>11 GPa), high temperature stability (>2000 °C), high electrical resistivity (>10^10^ Ω·cm), good thermal conductivity (>100 W/(m·K)), and good corrosion resistance [10]. The influences of AlN film thickness on SAW velocity and electromechanical coefficients have been explored in this work. Additionally, the temperature coefficients of frequency and quality factor of the SAW sensors have been measured from room temperature to 600 °C, thereby enabling the optimization of SAW device design for operation in harsh environments.

## 2. Experimental Setup

**Figure 1:**
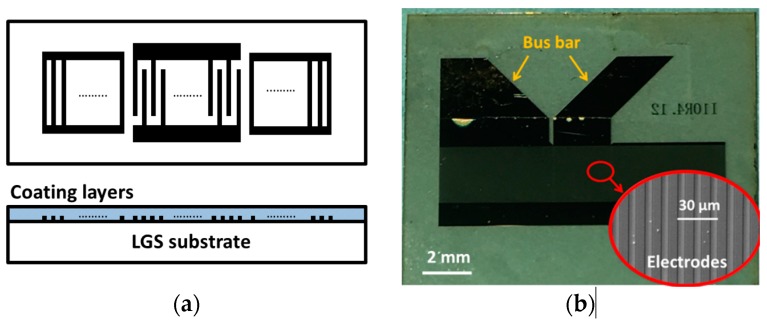
(**a**) Schematic illustration of one-port surface acoustic wave (SAW) resonator. The upper one is the top view and the lower one is the sectional view. LGS: langasite. (**b**) Photo of the prepared SAW resonator. Inset: SEM photo of the electrodes.

The LGS substrate with the Euler angles (0°, 138.5°, 26.6°) used in this paper were purchased from SICCAS, Shanghai. The SAW sensors had a typical one-port SAW resonator structure which consisted of an interdigital transducer (IDT) and two reflector banks. Each IDT contained 101 equal-interval-finger electrodes with finger widths of 6 μm, yielding an acoustic wavelength λ of 24 μm. Each reflector bank contained 400 short-circuited gratings. The aperture was 100 λ. The electrodes, which consisted of a 10 nm-thick Ti adhesion layer and a 100 nm-thick Au film, were patterned by lift-off photolithography techniques on LGS substrates. The protective AlN films were deposited on the surface of the SAW sensor by mid-frequency (40 kHz) reactive magnetron sputtering, except for on the electrode pad. The sputtering target was an aluminum disk with purity of 99.999%, whereas argon with purity of 99.999% was used as plasma gas and nitrogen with purity of 99.999% was employed as reactive gas. Before deposition, the sputtering chamber was evacuated to 1 × 10^−4^ Pa. After a 15-minute pre-sputtering on the Al target, N_2_ and Ar were both introduced into the chamber at a fixed flow ratio of 52 sccm:78 sccm. The sputtering power was 1900 W. The depositing rate of AlN overlayers was about 12 nm/min. In this work, the thickness of AlN films was in the range of 50 nm to 200 nm. The thicknesses of the AlN films were measured by a profile meter (Dektak 150, Veeco, New York, NY, USA). Before measurement, all of the samples were annealed at 600 °C for 30 min in pure N^2^ to improve their thermal stability. The thermal annealing process can not only improve the electrical properties of the Au/Ti electrodes at high temperature [11], but also eliminate voids in AlN coatings, so as to improve the thermal stability of the prepared LGS SAW sensor. The schematic illustration of the SAW resonator and the photos of the prepared SAW device are presented in Figure 1.

The prepared SAW devices were electrically characterized in air atmosphere using a vector network analyzer (VNA, Agilent E5071C, Agilent Technologies Inc., Santa Clara, CA, USA). The reflection scattering parameters (S_11_) of the prepared SAW devices were recorded and processed by a computer. In the experiment, the prepared SAW devices were continuously measured from room temperature to 600 °C for a period of 800 min. The resonance frequency of the SAW devices was recorded in 30 s intervals.

## 3. Results and Discussion

**Figure 2:**
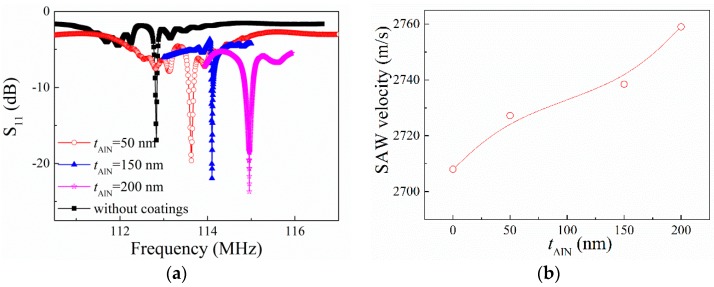
(**a**) S_11_ parameters of the SAW devices coated with AlN layers. The thickness of the AlN films is 0 nm, 50 nm, 150 nm, and 200 nm, respectively. (**b**) Dependence of experimental phase velocity on the thickness of AlN coating layers. The line is drawn as a guide for the reader.

The measured frequency responses of the prepared SAW resonators with different thickness of AlN coating layers (*t*_AlN_) are shown in Figure 2a. We can observe a clear resonance peak for every device. The resonance frequency increased from 112.8325 MHz to 114.9585 MHz when the *t*_AlN_ varies from 0 to 200 nm. The SAW phase velocity *v* can be calculated by

(1) $$v=\lambda \times f_{r},$$

where *f*_r_ is the resonance frequency of the SAW device obtained from the S_11_ curve. The calculated SAW velocities as a function of *t*_AlN_ are shown in Figure 2b. We can find that the SAW velocities increase with increasing *t*_AlN_. This is due to AlN guiding layers offering higher acoustic wave velocity (about 5100–5600 m/s [12,13]) than the LGS substrate (about 2700 m/s [14]). More acoustic wave energy will be concentrated in the guiding layers with increasing *t*_AlN_.

**Figure 3:**
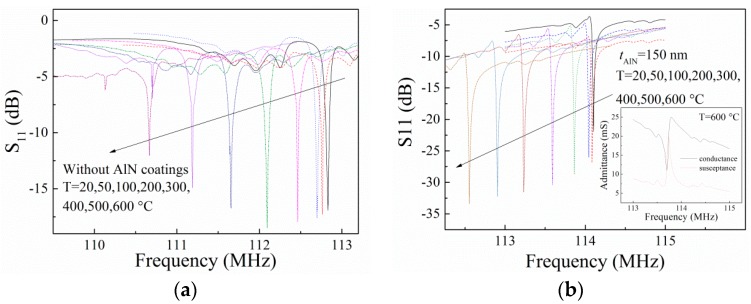
S_11_ results of (**a**) the bare LGS SAW devices without AlN coatings; and (**b**) the SAW devices coated with a 150 nm-thick AlN film at 20, 50, 100, 200, 300, 400, 500, and 600 °C. Inset: measured admittance (conductance and susceptance) of the SAW device when T = 600 °C.

The SAW devices without and with AlN coatings were measured from room temperature to 600 °C, and the S_11_ results of the SAW devices are shown in Figure 3. It can be found that the sharp resonance peak still exists at 600 °C, indicating that both of the SAW devices can work up to 600 °C. We also found that the resonance frequency decreases with increasing temperature. It can be interpreted as that both the LGS substrate and the AlN coating layer have a negative temperature coefficient of SAW velocity [14,15], which results in a decrease of the *f*_r_ of the SAW devices with increasing temperature. From Figure 3a,b, it can be observed that the height of the resonance peak of the SAW device without AlN coatings decreases at high temperature, while that of devices with AlN coatings do not attenuate at high temperature. This result suggests that the SAW device is indeed protected by the AlN coating layer at high temperature. With the measured S_11_ curve, the admittance (conductance and susceptance) of the SAW devices can be calculated, which is shown in the inset of Figure 3b. The admittance will next be used to calculate the electromechanical coupling coefficient (*K*^2^).

**Figure 4:**
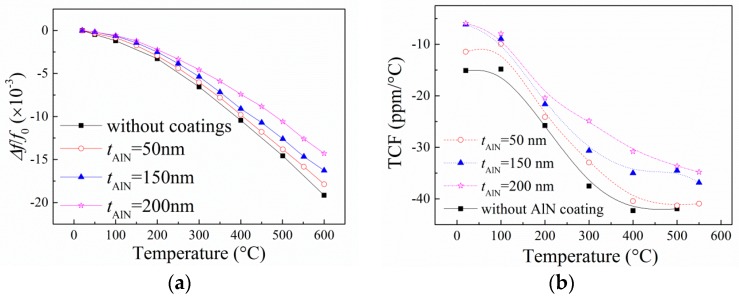
(**a**) Relative resonance frequency shift of the prepared SAW devices as a function of temperature. (**b**) Temperature coefficient of frequency (TCF) of the prepared SAW devices. The thickness of the AlN coatings is 0 nm, 50 nm, 150 nm, and 200 nm, respectively. The line is drawn as a guide for the reader.

The relative resonance frequency change (Δ*f*/*f*_0_) is defined as

(2) $$\Delta f/f_{0}=\frac{f_{T}-f_{0}}{f_{0}},$$

where *f*_T_ and *f*_0_ are the resonance frequency of the SAW devices at temperature T and room temperature, respectively. The Δ*f*/*f*_0_ for the SAW devices with different thickness of AlN coating layers as a function of temperature is presented in Figure 4a. It can be observed that the Δ*f*/*f*_0_ is dependent on the AlN film thickness. The absolute value of Δ*f*/*f*_0_ decreases with increasing AlN film thickness at the same temperature.

Experimentally, the temperature coefficient of frequency (TCF) can be extracted from the relative shifts of the resonance frequency by

(3) $$\mathrm{TCF}=\frac{1}{f_{r}}\frac{\partial f_{r}}{\partial T}.$$

It can be observed that the absolute TCF value (|TCF|) of the devices (i.e., the slope of the curve at each temperature) increases with the increasing temperature, as shown in Figure 4b. For example, the |TCF| of the SAW device with a 200 nm-thick AlN coating layer is about 5.2 ppm/°C at 25 °C, while it increases to 34 ppm/°C at 600 °C. When temperature is higher than 400 °C, the TCF value approximates to a constant—i.e., the resonance frequency of the SAW devices decreases quasi-linearly with increasing temperature. The temperature behaviors of the SAW devices are also dependent on the thickness of the AlN coating layer. It can be found that the |TCF| decreases with increasing *t*_AlN_. This is due to the fact that the coefficient of thermal expansion (CTE) of the AlN film is smaller than that of the LGS substrate [16,17]. Therefore, the effective CTE of the AlN film-coated LGS SAW devices would decrease with increasing *t*_AlN_, thereby causing a reduction of the |TCF| of the SAW devices. On the other hand, more acoustic wave energy would be concentrated in the AlN films with increasing *t*_AlN_, resulting in a decrease in |TCF|, approaching the value of AlN films (about −30 ppm/°C [12]), because the |TCF| of AlN is smaller than that of the LGS material at high temperature (about −48 ppm/°C at 500 °C [5]).

**Figure 5:**
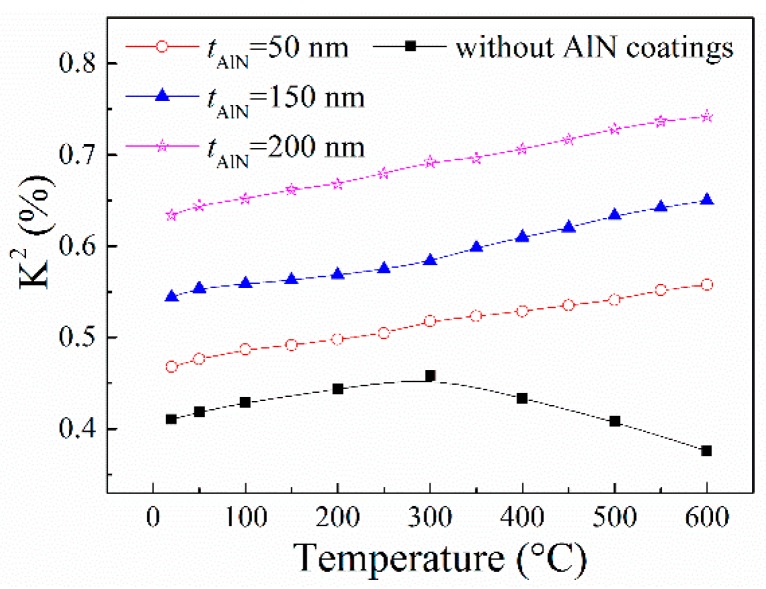
Electromechanical coupling coefficient *K*^2^ of the SAW devices with different thickness of AlN coating layers as a function of temperature.

The *K*^2^ of the SAW devices can be calculated by [18]

(4) $$K^{2}=\frac{\pi }{4N}\frac{G_{m}\left(f_{r}\right)}{B_{s}\left(f_{r}\right)},$$

where *N* is the number of IDT finger pairs and equals to 100 in this work, *f*_0_ corresponds to the resonance frequency, and *G*_m_(*f*_r_) and *B*_s_(*f*_r_) are the motional conductance and static susceptance of the device at *f*_r_. The calculated *K*^2^ as a function of temperature is plotted in Figure 5. It can be found that the *K*^2^ of the SAW device without AlN coatings increases firstly with increasing temperature and decreases with further increase of temperature. We think that the decrease of *K*^2^ at high temperature is due to the degradation of metal electrode at high temperature [19]. We also find that the *K*^2^ of the SAW devices coated with AlN films increases with increasing temperature, and the *K*^2^ increases by about 20% from room temperature to 600 °C. These results also suggest that the AlN film can be used as a good protective layer. Moreover, the *K*^2^ increases with increasing *t*_AlN_—i.e., the SAW device would have better performance with thicker AlN coatings. However, with further increase of the *t*_AlN_, the AlN coatings would have negative effects on the properties of the LGS SAW devices due to mass effect, leading to reduction of the resonance strength of the SAW devices [20].

## 4. Conclusions

In this work, the temperature behaviors of the LGS SAW devices coated with AlN thin films have been investigated. The AlN films with different thickness were deposited on bare LGS SAW devices by mid-frequency magnetron sputtering. The results show that the performance of the bare SAW device without AlN coatings attenuates to a certain extent at high temperature, while the SAW devices coated with AlN films show good performance even at 600 °C. The SAW velocity increases with increasing thickness of the AlN coating layers. The resonance frequency of the prepared SAW device decreases with increasing temperature, from room temperature to 600 °C. The *K*^2^ of the SAW devices coated with AlN films increases with increasing temperature, which means that these SAW devices have good performance at high temperature. It can be concluded that the AlN coating layer can not only protect the SAW devices from environmental contamination, but also improve their performance. The results of this work suggest that the SAW devices with AlN coating layers are suitable for high temperature sensing applications. However, it should be noted that the thickness of the AlN coating layer should be optimized if one expects to use the SAW device as a temperature sensor with large *K*^2^ and large |TCF|, because the AlN coatings may reduce the |TCF| of the LGS SAW devices.
